# Using Motivational Interviewing to reduce threats in conversations about environmental behavior

**DOI:** 10.3389/fpsyg.2015.01015

**Published:** 2015-07-21

**Authors:** Florian E. Klonek, Amelie V. Güntner, Nale Lehmann-Willenbrock, Simone Kauffeld

**Affiliations:** ^1^Department of Industrial/Organizational and Social Psychology, Institute of Psychology, Technische Universität BraunschweigBraunschweig, Germany; ^2^Department of Experimental and Applied Psychology, Faculty of Psychology and Education, VU University AmsterdamAmsterdam, Netherlands

**Keywords:** Motivational Interviewing, environmental behavior, intervention study, interaction analysis, self-determination theory

## Abstract

Human behavior contributes to a waste of environmental resources and our society is looking for ways to reduce this problem. However, humans may perceive feedback about their environmental behavior as threatening. According to self-determination theory (SDT), threats decrease intrinsic motivation for behavior change. According to self-affirmation theory (SAT), threats can harm individuals’ self-integrity. Therefore, individuals should show self-defensive biases, e.g., in terms of presenting counter-arguments when presented with environmental behavior change. The current study examines how change recipients respond to threats from change agents in interactions about environmental behavior change. Moreover, we investigate how Motivational Interviewing (MI) — an intervention aimed at increasing intrinsic motivation — can reduce threats at both the social and cognitive level. We videotaped 68 dyadic interactions with change agents who either did or did not use MI (control group). We coded agents verbal threats and recipients’ verbal expressions of motivation. Recipients also rated agents’ level of confrontation and empathy (i.e., cognitive reactions). As hypothesized, threats were significantly lower when change agents used MI. Perceived confrontations converged with observable social behavior of change agents in both groups. Moreover, behavioral threats showed a negative association with change recipients’ expressed motivation (i.e., reasons to change). Contrary to our expectations, we found no relation between change agents’ verbal threats and change recipients’ verbally expressed self-defenses (i.e., sustain talk). Our results imply that MI reduces the adverse impact of threats in conversations about environmental behavior change on both the social and cognitive level. We discuss theoretical implications of our study in the context of SAT and SDT and suggest practical implications for environmental change agents in organizations.

## Introduction

Most of us have an understanding that natural and energy resources are finite and have considered our own carbon footprint. However, even if we assume that people today are generally aware of the importance of pro-environmental behavior, there is still a considerable discrepancy between committing to pro-environmental norms and the actual behavior that contributes to environmental protection (Séguin et al., 1998; Vining and Ebreo, 2002; Castro et al., 2009; Fritsche et al., 2010).

A possible explanation for this discrepancy can be derived from the notion that confrontation with one’s own poor environmental behavior and its expected consequences can elicit individual perceptions of threat. The intention behind many pro-environmental initiatives is that individuals will respond to negative feedback about their environmental behavior by changing their behavior and becoming more environmentally friendly. However, people often resist such feedback and therefore lack positive behavior change in terms of more sustainable activities (Sherman and Cohen, 2002, 2006). Information about climate change is potentially threatening to individuals because it implies changes and constraints in human living conditions (Fritsche et al., 2012). More generally speaking, “threats result from some experience of discrepancy between an expectation or desire and the current circumstance” (Jonas et al., 2014, p. 229).

In order to alleviate individual responses to threats in social interactions, we introduce Motivational Interviewing (MI) as a communication approach. We explicate how MI can prevent potential threats in social interactions about behavior change by reducing the actual amount of threats (social effect) as well as alleviating the negative perception of threats (cognitive effect). Furthermore, we argue for an interplay between change agents’^1^ verbal behavior and the verbally expressed motivation of their change recipients.

### Confrontational Behavior: Threats in Social Interactions

A substantial body of research has focused on various reasons why individuals may experience threats in social interactions (e.g., Festinger, 1957; Brehm, 1966, 1993; Steele, 1988; Jost and Banaji, 1994; Blascovich and Mendes, 2000; Gawronski, 2012; Jonas et al., 2014). Means by which people feel threatened are, for example, verbal confrontations with a certain discrepancy at present. Previous research argues that the experience of discrepancy between a desired state and the actual situation entails challenges to the fulfillment of individuals’ psychological needs (Jonas et al., 2014).

To understand sources of threat in social interactions, one research stream highlights individuals’ need for self-relevant clarity and cognitive consistency (Hogg, 2007; Swann, 2011), while other studies have focused on individuals’ need for self-worth or self-integrity (e.g., Tajfel and Turner, 1979; Steele, 1988). Other studies have focused on the overall need for order and stability. For example, system justification theory (Jost and Banaji, 1994) states that individuals have the need to create and maintain a favorable self-image, also labeled as “ego justification.” This justification tends to occur at the expense of others (e.g., group interests) and undermines individuals’ motivation to change their behavior or attitude (Jost and Banaji, 1994; Jost et al., 2010).

A second stream of research emphasizes individuals’ need for personal control (Kay et al., 2009; Leotti et al., 2010), behavioral autonomy (Deci and Ryan, 2000, 2002), and freedom in general (Brehm, 1966, 1993). For example, Brehm’s (1966, 1993) theory of psychological reactance illustrates that individuals believe that they hold the freedom to engage in behaviors as they please — based on the satisfaction of their needs. When this perceived behavioral freedom is threatened by a persuasive message, individuals are motivated to reinstate that particular freedom. Consequently, they engage in defensive behaviors that are directed away from the behavior that the persuasive message targets on. This effect has also been labeled as the “boomerang effect” (Brehm, 1966, 1993).

Based on these previous findings and in line with Jonas et al. (2014), we conclude that the experience of discrepancy and unfulfilled personal needs can be considered a primary source of threat. We apply this assumption to the case of feedback about environmental behavior: That is, individuals who are confronted with a discrepancy between their current environmental behavior and a desired pro-environmental behavior, experience this as a threat. Seen through a social psychology lens, we offer the following possible explanations underlying subsequent defensive responses.

### Self-Defense and Motivational Responses from a Social Psychology Perspective

There are several reasons why negative feedback about environmental behavior may elicit perceptions of threat. First, such negative feedback can threaten an individual’s need for self-integrity, because the suggestion that one has harmed the environment (e.g., by littering or by leaving the lights on) conflicts with the belief about being an environmentally conscious person. According to self-affirmation theory (SAT; Steele, 1988), individuals have a fundamental motivation to protect their personal image of self-integrity, in terms of seeing themselves as moral, adaptive, and capable of controlling important outcomes. When confronted with self-threatening information, individuals show self-defense responses such as denying threatening information, presenting counter-arguments, or expressing resistance to change in order to restore their self-integrity (Sherman and Cohen, 2002, 2006).

Second, individuals may perceive negative verbal feedback that asks for environmental behavior change as a threat to their autonomous decision-making ability (Osbaldiston and Sheldon, 2003). According to self-determination theory (SDT; Deci and Ryan, 2000, 2002; Ryan and Deci, 2000, 2002), individuals can experience motivation and self-determination in their behaviors only when the need for autonomy is fulfilled (Ryan et al., 1995; Deci and Ryan, 2000, 2002; Ryan and Deci, 2000, 2002). SDT argues that humans have a natural interest in pursuing self-determined goals and behaviors, rather than pursuing goals directed by external forces. In line with SDT, we assume that the development of motivation is strongly dependent on social context, such as the relevant interaction partners (Deci and Ryan, 1987). In particular, we argue that individuals may experience feedback about their environmental behavior as a potential threat to their autonomy and self-determination. As a result, individuals are less likely to develop the necessary motivation to respond with environmental behavior change. There is preliminary support for this general idea in environmental psychology research. For example, Osbaldiston and Sheldon (2003) asked their participants to commit to pro-environmental goals. The authors reported participants’ perceptions of the experimenters’ autonomy-supportive behavior were positively linked to greater internalized motivation.

In sum, we conclude that individuals may react with defensive responses in order to restore their psychological needs when they experience a discrepancy between a desired and their current behavior (Jonas et al., 2014). However, in the face of necessary environmental behavior change, such defensive responses are problematic. When individuals resist threatening environment messages, positive behavior change in terms of more sustainable activities becomes unlikely (Sherman and Cohen, 2002, 2006). This highlights the challenge of bringing about environmentally friendly behavior change. What is needed to address this challenge is a means to create circumstances under which individuals do not perceive negative feedback about their environmental behavior as a threat to their self-integrity as well as to their perceived autonomy.

### Reducing Threats: The Case for MI

As one such means, we introduce the intervention method of MI. When considering conversations about environmental behavior change, MI can provide a practical skill that helps preserve the self-integrity and autonomy of the conversational partner (e.g., Markland et al., 2005; Vansteenkiste and Sheldon, 2006; Leffingwell et al., 2007; Vansteenkiste et al., 2012). MI is defined as a “collaborative conversation style for strengthening a person’s own motivation and commitment to change” (Miller and Rollnick, 2013, p. 12). Although it remains to be seen how MI fares in the context of environmental behavior change, several meta-analytic studies have established MI as an evidence-based intervention method in facilitating behavior change in the clinical context (e.g., Hettema et al., 2005; Rubak et al., 2005; Carroll et al., 2006; Lundahl et al., 2010; Magill et al., 2014).

We have outlined that individuals may perceive feedback about their environmental behavior as threatening. MI may alleviate this tension by asking change agents to abstain from confrontations or from trying to impose strategies for behavior change (Miller and Rollnick, 2013). In other words, we argue that MI prevents change recipients from threats to their self-integrity. MI provides methods to deal with client resistance and to support clients’ self-efficacy by validating negative client statements (Werner et al., 2009) and by selectively attending to clients’ verbal expressions in favor of change (i.e., change talk, Miller and Rose, 2009; Glynn and Moyers, 2010). Change talk might include statements such as “There are certainly some options to save energy” or “I am going to implement this right away.” By contrast, sustain talk captures clients’ verbal expressions against change, such as “It is just not so simple, not while I have all my work to do” or “But that’s the way we always did it.”

Furthermore, individuals may experience feedback about (poor) environmental behavior as a potential threat to their perceived ability in making autonomous decisions. MI addresses this need for autonomy through the belief that the recipient, rather than the change agent, is the primary source of ideas and solutions for accomplishing behavior change. Accordingly, change agents should serve the need for autonomy in deciding about solutions for reaching these goals. Moreover, more autonomously regulated behaviors are not only executed more persistently, but also with greater care and quality (Ryan and Deci, 2000).

In sum, we argue that MI as a communication method serves the fulfillment of psychological needs, particularly the need for self-integrity and autonomy (e.g., Steele, 1988; Deci and Ryan, 2000, 2002; Vansteenkiste and Sheldon, 2006; Leffingwell et al., 2007). MI can create the very conditions that help reduce perceived threat to self-integrity and autonomy when individuals are confronted with negative feedback. As a result, MI is likely to foster individuals’ motivation to engage in pro-environmental behavior.

Motivational Interviewing as a communication method has been applied in numerous behavior change settings, such as reducing risk behavior (e.g., Colby et al., 1998; McCambridge and Strang, 2004), treating psychological problems (Burke, 2011), or promoting healthy behavior (cf., Lundahl et al., 2010; Perry and Butterworth, 2011). Whereas MI was traditionally taught to practitioners in the helping professions, it is also highly suitable for the context of environmental behavior (Tribble, 2008; Forsberg et al., 2014; Klonek and Kauffeld, 2015). For example, Klonek and Kauffeld (2015) suggest that human resources departments could provide MI training for energy managers in order to reduce organizational energy consumption. In support of this argument, Forsberg et al. (2014) showed that MI training increased the conversational skills of Swedish environmental inspectors. Furthermore, the authors showed that MI training positively affected inspectors’ empathy (rated by external observers).

However, several gaps in the literature remain. First, whereas the assessment of empathy by means of independent observers aligns with most previous research in the context of MI applications (e.g., Catley et al., 2006; Tollison et al., 2008; Forsberg et al., 2014), this approach tends to neglect the actual perspective of the change recipients. In other words, change recipients should be best suited to evaluate whether their interactional partner has confronted them or demonstrated empathic understanding. We aim to address this gap in the literature by considering both social and cognitive reactions by change recipients. A second gap concerns the lack of human interaction in some previous work on MI in the context of environmental behavior. Tribble (2008) conducted a laboratory computer experiment in which participants performed a decisional balance task – which is typical of a MI intervention – that is, they listed pros and cons about changing their environmental behavior. The intervention had no effect on the environmental outcome scores. Tribble (2008) attributed this to the fact that the MI task was realized within a computer environment, that is, it lacked the expression of empathy by a real human being. A third gap in the literature (e.g., considering the study by Forsberg et al., 2014) is the lack of data on client verbal responses (in terms of change talk and sustain talk). Taken together, we aim to address these gaps in the literature (1) by examining a human interaction-based MI intervention that focuses on environmental behavior change, (2) by considering both agent and recipient behavior within the conversation process, and (3) by including cognitive reactions to the change conversation, in terms of change recipients’ perceptions about confrontation and empathy of change agents.

### Hypothesis Building

#### Social Effects of MI

Avoiding autonomy-restrictive or verbal threats is one of the core principles in the application of MI (e.g., Miller et al., 2004). MI advises not to confront change recipients with direct argumentation or suggestions for change, but instead to encourage them to develop their solutions (Miller and Rollnick, 2013). Hence, we expect that MI should become expressed in terms of the actual verbal behavior of change agents. Specifically, change agents using MI should show fewer verbal behavioral threats such as suggestions, confrontations, or argumentations about behavior change toward more environmentally conscious behavioral conduct. In other words, we expect that feedback by change agents who use MI involves a lower amount of actual behavioral threats than feedback by change agents who do not use MI. Concerning the social effects of MI skills, we hypothesize the following:

H1: Using MI in environmental feedback conversations reduces the amount of verbal threats by change agents.

#### Cognitive Effects of MI

In addition to avoiding autonomy-restrictive behavior, showing empathic behavior has been considered just as important in the use of MI and for effective communication in general (Miller and Mount, 2001; Miller et al., 2004; Miller and Rose, 2009; Moyers and Miller, 2013). Previous research has demonstrated the relevance of empathy for perceptions of safety and trust in physician–patient relationships and, in turn, for the quality of conversational outcomes (e.g., Miller et al., 1980; Hojat et al., 2002; Moyers et al., 2005; Nicolai et al., 2007; Gaume et al., 2009). Although these previous findings were obtained in clinical settings, we assume that the core value of empathic communication can apply in the context of environmental feedback as well.

Empathy can be considered a multidimensional construct that can function on either a cognitive or an affective level (Davis, 1983; Galinsky et al., 2008; Reniers et al., 2011). The affective component of empathy refers to the capacity to show appropriate emotional reactions toward other people and to the experiences they articulate. The cognitive component of empathy describes the ability of an individual to perceive the world from another person’s perspective by putting oneself in that person’s position (Davis, 1983; Reniers et al., 2011).

In the context of MI, empathy is served by means of verbally communicating empathic understanding through reflective listening. Reflective listening means that change agents paraphrase verbal statements of their conversational partners (also termed empathic back-channeling; Miller and Rollnick, 2013). That is, change agents need to put themselves in the position of the recipient. Previous studies suggest that putting oneself in the position of another person, i.e., perspective taking, improves the relationship that is necessary for collaboratively reaching solutions (e.g., Parker and Axtell, 2001; Galinsky et al., 2008; Steindl and Jonas, 2012). Thus, change recipients should experience a conversation with MI change agents as highly empathic.

Further, we can assume that reflective listening in social interactions helps change recipients pay attention to their own argumentation (for behavior change) instead of being pushed toward pro-environmental behavior by a change agent (cf., Hettema et al., 2005). For example, instead of saying “You should realize that your behavior harms the environment,” change agents using MI might reflect a previous recipient statement as “You mentioned that you realized that your behavior harms the environment.” Even though, the content of both sentences in this example might impose a threat (i.e., a discrepancy between recipients’ current behavior and desired behavior), the MI conversational style should alleviate change recipients’ perception of threats during a conversation. Consequently, change recipients’ cognitions about being confronted about their environmental feedback should be reduced through the use of the MI conversational style.

H2: Using MI in environmental feedback affects change recipients’ cognitions about the social interaction in terms of (a) increasing perceived empathy and (b) decreasing perceived confrontation.

#### The Interplay between Social Behaviors and Cognitions

Further, the present study aims at gaining insight into the interrelation between verbal behaviors of change agents and cognitions of change recipients in social interactions about behavior change. The underlying mechanism is that change agents interpersonal behaviors within the social interaction shape clients’ cognitions in a way that also affects their motivation to change a specific behavior. Specifically, we expect that change agents who use MI demonstrate fewer threats when giving feedback about environmental behavior. As a result, change recipients should perceive MI-feedback as less confrontational. The link between change agents’ social behavior and change recipients’ social cognitions can prevent negative conversational dynamics that are typically evoked by confrontational change agents (Miller and Rollnick, 2002; Klonek et al., 2014). In sum, we argue for an interplay between change agents’ verbal threats and clients’ corresponding cognitions. Put formally:

H3: Change agents’ social behavior and recipients’ perceptions of agents’ behavior are intertwined, such that the amount of observed threats is positively related to perceived confrontation and negatively related to perceived empathy.

#### SAT and SDT based Predictions of Threats in Conversations about Environmental Behavior

We have outlined in a previous section how SAT and SDT can help explain interpersonal dynamics in social interactions about behavior change. Next, we derive predictions regarding agent–recipient dynamics from both theories.

**FIGURE 1:**
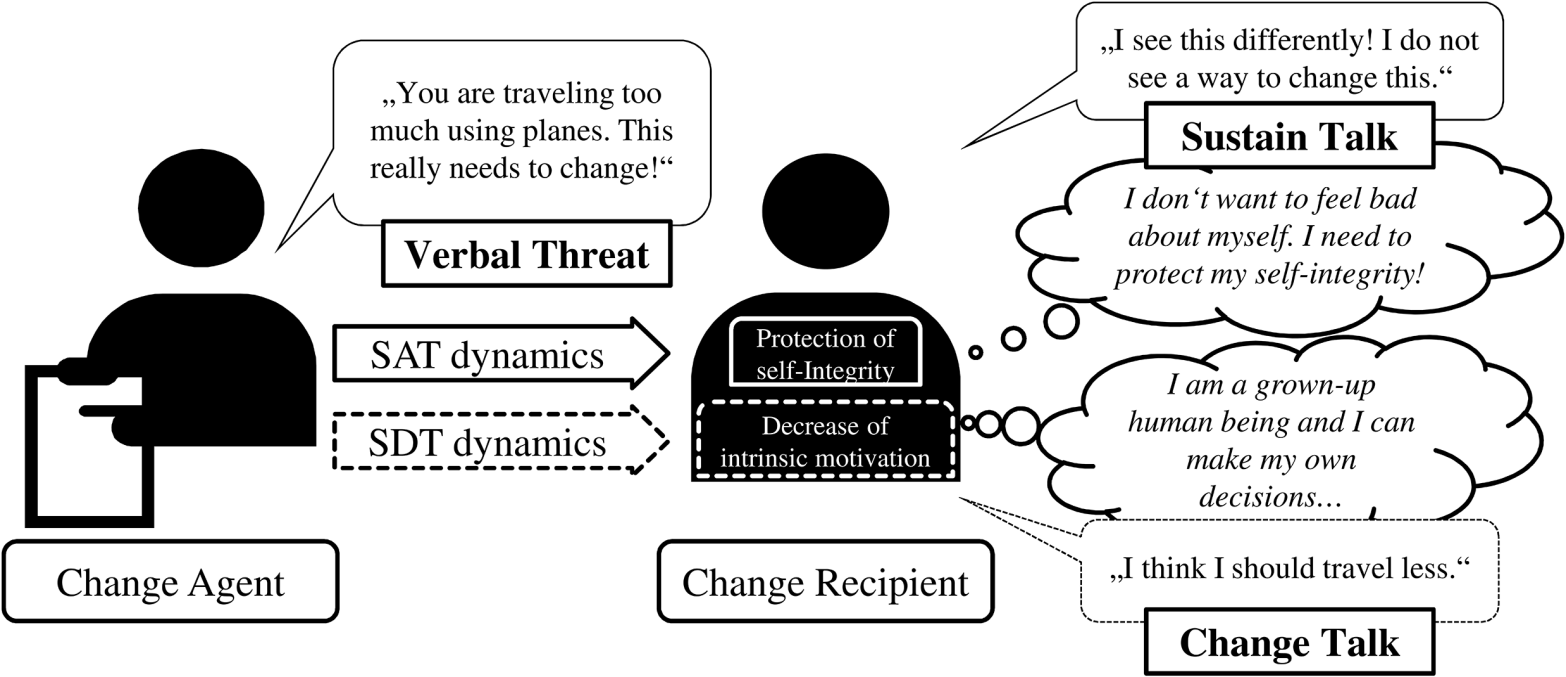
**Illustrative example of how SDT and SAT predict how verbal threats affect social interactions.** Facilitative effects = “—,” Inhibitory effects = “-----.” The upper line shows predictions based on SAT for the social interaction and the lower line shows predictions for SDT. SAT, self-affirmation theory; SDT, self-determination theory.

Based on SDT (Deci and Ryan, 2000, 2002; Ryan and Deci, 2000, 2002), change agents should address recipients’ need for autonomy in order to evoke intrinsic motivation. The less change recipients are threatened with behavior change, the more likely they will make self-determined decisions about behavior change. The interpersonal dynamics derived from SDT are depicted in **Figure 1**. Verbal threats negatively affect self-determination of participants, that is, verbal threats that feedback a gap between actual and desired environmental behavior will harm the intrinsic motivation of change recipients. As illustrated in **Figure 1**, on the cognitive level, change recipients might have thoughts during the interaction such as “I’m an adult and I can make my own decisions.” As a result, change recipients should be less likely to show change talk on the observable interpersonal behavior level. In other words, change recipients should be less likely to provide change-directed utterances such as “I think I should travel less” that indicate that they have their own reasons for change (Miller and Rose, 2009; Glynn and Moyers, 2010). Therefore, we expect that:

H4: Change agents’ verbal threats will be negatively related to change recipients’ motivation (i.e., change talk).

Different from SDT, SAT (Steele, 1988) posits that threats about behavior change are negatively affecting change recipients’ self-integrity or self-esteem (see **Figure 1**). In order to protect their self-esteem, change recipients can use self-defense strategies such as counter-arguments, denying threatening information, or expressing resistance to change (Sherman and Cohen, 2002, 2006). That is, based on SAT, we assume that change recipients who are threatened with behavior change are more likely to protect themselves using counter-change language (i.e., self-defenses). In MI, counter-change language is captured in terms of sustain talk (Miller and Rose, 2009; Glynn and Moyers, 2010). An example of counter-change language that change recipients could use to defend their self-integrity would be “I see this differently! I do not see a way to change this.” We hypothesize:

H5: Change agents’ verbal threats will be positively related to change recipients’ sustain talk.

Meta-analytic findings on the use of MI in clinical settings provide initial evidence for these predictions derived from SDT on the one hand and SAT on the other hand. Magill et al. (2014) found that verbal threats (i.e., MI non-adherent behavior) were linked to an increase of sustain talk and a decrease of clients’ motivation in terms of change talk. Although this interplay between change agents’ and recipients’ verbal behaviors has not yet been examined in the context of environmental behavior change to date, we assume similar behavioral linkages in this domain.

### Current Research

In order to test our hypotheses, we compared interpersonal (i.e., verbal behavior) and cognitive variables (i.e., change recipients’ perceptions of confrontation and empathy) between two groups of dyadic change-related conversations. In each dyad, change agents discussed discrepancies between current environmental behavior and ideal pro-environmental behavior. In one group of dyads, change agents applied MI (intervention group), whereas the other group of change agents served as a control group. Control change agents were instructed to give change recipients feedback on their environmental behavior and motivate them to improve their behavior.

The data for this study were gathered as part of a larger research project. We have reported detailed information about the reliability of our observational coding instrument in a previous publication (Klonek et al., 2015b).

In the present study, we investigated how verbal threats of social change agents differed between the two social-interaction based approaches (H1). To do so, we enumerated the frequencies of change agents’ observed MI non-adherent behaviors and compared these between the MI and control group.

We also investigated how verbal threats (i.e., MI non-adherent behavior) in both groups affect social cognitions of change recipients (H2 and H3). To do so, we measured change recipients cognitive reactions after the interview.

Moreover, we were interested in the interpersonal dynamics between change agents’ verbal threats and change recipients’ motivation and self-defense (H4 and H5). Therefore, we also counted change recipients’ instances of change and sustain talk during the conversation and related the frequency of these instances to change agents’ social behavior.

Finally, we also measured environmental attitude and environmental intentions using a validated survey instrument (SEU-3; Schahn, 1999 and Schahn et al., 2000) before and after the intervention, in order to investigate whether the MI intervention positively affected environmental outcome measure in comparison to the control group.

## Materials and Methods

### Sample

We excluded nine conversations from our analysis because change recipients in those dyads did not provide self-report data on empathy and confrontation. Our final sample contained 68 dyadic conversations about environmental behavior change. Twenty-six participants took the role of a change agent. The majority of the change agents in the MI group were enrolled as psychology majors (*n* = 14); one change agent in the MI group was enrolled in human resources development. The average age of the MI agents was 29 (SD = 8.33). The gender ratio was balanced with seven male change agents. Change agents in the control group (*n* = 11) were 31 years on average (SD = 13.63). The gender ratio was balanced (six female, five male). Six of the change agents in the control group had a technical or natural science background, and five change agents had a background in psychology or the social sciences.

Change agents in the MI group (*n* = 15) received training in MI before they took part in this study. The MI training comprised 21 h over a period of 3 months as part of their psychology coursework. The training was designed according to the eight stages of learning MI (Miller and Moyers, 2006) and contained exercises from the Motivational Interviewing Network of Trainers (2008) manual.

Change agents in both groups had a conversation with a change recipient about pro-environmental behavior change. All change agents were instructed to motivate their change recipients to increase pro-environmental behavior and to work out individual measures that participants should implement. In order to include a manipulation check of our intervention, change agents were asked to indicated their level of familiarity and proficiency in MI on a five-point Likert-type scale ranging from 1 (strongly disagree) to 5 (strongly agree). Change agents in the MI group scored significantly higher on familiarity with (M = 3.79 vs. M = 1.6, *p* < 0.01) and proficiency in MI (M = 2.93 vs. M = 1.8, *p* < 0.01), in comparison to change agents from the control group.

For a second and more objective manipulation check of our intervention variable, we also assessed change agents’ MI spirit using a rating scale from the German MITI-d (MI Treatment Integrity; Brueck et al., 2009). The MI spirit scale assesses the overall skillfulness in using MI and indicates the extent to which the change agents in our sample showed evocation, collaboration and autonomy within the conversation. External observers were asked to estimate change agents’ overall adherence to MI principles (Moyers et al., 2010).

Two observers rated the extent to which change agents showed MI spirit on a seven-point scale (1 = weak adherence, 7 = strong adherence). The extremes of the rating scales were verbally anchored with the definition of strong/weak MI spirit adherence. ICCs and Cronbach’s α were used to estimate inter-rater reliability for this measure (ICC = 0.76 and α = 0.87). Change agents in the MI group received significantly higher MI spirit adherence scores (M = 5.77) in comparison to change agents from the control group [M = 4.28, *t*(23.98) = -5.67, *p* < 0.01]. A value of 5 and higher on the MI spirit adherence scale is considered as basic proficiency in MI, whereas a value of 6 and higher is considered as solid proficiency in MI (Brueck et al., 2009).

Change recipients (*n* = 68) were 24 years old on average (SD = 7.83) and the majority was female (78%, *n* = 53). Most of them had a high school degree (76.5%, *n* = 52), 15% (*n* = 10) had a university degree, 7.4% had finished a vocational training (*n* = 5), and 1.5% (*n* = 1) held at least a secondary school-level education. Age, gender, prior vocational training, and educational level did not differ significantly between the MI and the control group. Prior to the conversation, participants in both groups reported their environmental behavior on a 28-item environmental behavior scale by Schahn (1999) and Schahn et al. (2000); e.g., “I only use energy-saving devices in order to save electric energy”) on a five-point response format (1 = strongly disagree to 5 = strongly agree). We found no differences across the two groups regarding this measure (M = 3.36 in the MI and control groups, *p* = 0.96).

### Procedure

Prior to data gathering, the study protocols were approved by the institutional review board for data security. All participants gave informed consent for videotaping their conversations. Based on their availability, change recipients were allocated to a conversation with either a change agent in the MI group (*n* = 49) or in the control group (*n* = 19). Change agents and recipients were unaware of the hypotheses of this study. Three sessions (two in the intervention and one in the control group) could not be recorded due to technical problems. Before each conversation started, the change recipients completed the self-report measure about their environmental behavior. The change agents received this information in order to talk about environmental behaviors that could be improved and a short written agenda that listed the following topics: setting the agenda, asking about current environmental behavior, giving feedback about environmental behavior to change recipients, asking for measures to increasing pro-environmental behavior, and planning measures/giving advice (see Appendices A–D for detailed information).

### Change Recipients’ Environmental Behavior, Attitude, and Intentions

We measured environmental behavior, attitudes, and environmental intentions using three validated 28-item measures from Schahn (1999) and Schahn et al. (2000). A sample item for environmental behavior was “I only use energy-saving devices in order to save electric energy.” A sample item for environmental attitude was “In my opinion, grocery shops still sell too many environmental harmful products.” A sample item for environmental intentions was “In the future, I will specifically ask for environmentally friendly products and ask the grocery store to change the assortment of goods accordingly.” Environmental attitudes and intentions were measured before and immediately after the conversation. Internal consistencies for environmental behavior (α = 0.70) was acceptable; internal consistencies for environmental attitude (α = 0.87–0.88) and environmental intentions (α = 0.88–0.86) were good (Kline, 2000).

### Environmental Action Plan

All change recipients had the opportunity to fill out a change plan worksheet after the interview (based on Kauffeld et al., 2009; Magill et al., 2010). This worksheet listed the sentence “I will carry out the following measures” followed by consecutively numbered opened spaces for writing down intended actions. The number of measures was summed up to derive a measure of “number of environmental actions.”

### Change Agents’ Verbal Threats

We measured verbal threats of change agents with an observational coding instrument from MI (i.e., the MITI-d, Brueck et al., 2009). The German MITI-d includes the behavior code “MI non-adherent behavior.” This behavior code encompasses autonomy-restrictive behaviors, such as “confrontations” (e.g., directly and unambiguously disagreeing, arguing, correcting, shaming, blaming, criticizing, labeling, moralizing, or ridiculing), “advising without permission” (making suggestions, offering unsolicited advice), and “directing” (e.g., giving orders, commands, or imperatives). Within conversations about environmental behavior change, MI non-adherent behaviors comprised feedback about discrepancies between a desired environmental behavior and the current circumstance (i.e., threats) using a tone of uneven power sharing, disapproval, or negativity.

### Change Recipients’ Social Reactions of Intrinsic Motivation and Self-Defense

We operationalized motivation and resistance to change (i.e., as a sign of self-defense) by coding change recipients’ verbal reactions during the videotaped conversations. In line with previous research (Miller et al., 2008; Klonek et al., 2015a), statements with a positive inclination toward change were coded as change talk (e.g., “When I am not home the entire day, I do not need the lights on.”), whereas statements that had a negative inclination toward change were coded as sustain talk (e.g., “In my opinion, changing my behavior will not make a difference,” **Table 1**). Verbal statements containing no apparent inclination toward or against change were coded as follow/neutral (e.g., “I don’t have a washing machine”). We further subclassified change and sustain talk utterances into reasons, activation/other, taking steps, and commitment to change or to sustain, respectively (cf., Miller et al., 2008). We used change talk codes as measures of verbally expressed motivation and sustain talk codes as measures of verbally expressed resistance and self-defense.

**Table 1:** Examples of change talk and sustain talk in the context of pro-environmental behavior.

	Change talk (+)	Sustain talk (-)
Reasons	When I am not home the entire day, I do not need the lights on.	In my opinion, changing my behavior will not make a difference.
Activation^1^	There are certainly some options to save energy.	I see it just is not so simple, not while I have all my work to do.
Taking steps	I have set up my PC with a coupler strip so that it is not running on standby the entire time.	Last week, I did not shut my laptop down while I was working in the kitchen.
Commitment	I am going to implement this right away.	I will not change this behavior in the future.

*^1^This code is labeled as “Other” in the MI skill code (Miller et al., 2008).*

In order to adjust for time differences between conversations, we standardized the frequencies for each behavioral code to a 10-minute interval (i.e., “rates”; cf. Bakeman and Quera, 2011, p. 96 and 101).

#### Inter-Rater Reliability

Two independent observers were extensively trained in classifying the verbal behaviors of change agents and change recipients. They received a set of graded learning tasks, including scripted interactions from the developer of the MITI-d, MI video material (Demmel and Peltenburg, 2006), and recorded demo-interactions for learning how to code MI-relevant behavior. To train for coding change recipients’ verbal behaviors, we provided additional transcripts and audio material (Project MILES, 2011). Further training material for training observers in using the MITI-d is also available online (cf., supplemental material in Klonek et al., 2015b). The videos were coded using INTERACT software (Mangold, 2010). For detailed psychometric information about the software-supported coding scheme, see Klonek et al. (2015b). A random sample of 13 interactions (19%) was coded twice. We calculated the Intraclass Correlations (ICC) for these sessions to obtain a measure of observer reliability for change and sustain talk measures. The ICC is a statistical index commonly used to estimate reliability because it adjusts for chance agreement and systematic differences between observers (Fleiss and Shrout, 1978; McGraw and Wong, 1996, p. 35). We classified all obtained ICC values according to the criteria proposed by Cicchetti (1994). Except for our measure of “sustain talk–taking steps” (ICC = 0.36), all behavioral codes in our sample showed fair to excellent inter-rater reliability (ICCs = 0.51–0.91).

### Cognitive Reactions

In terms of cognitive reactions to the change conversations, we measured change recipients’ perceptions of empathy and confrontation immediately after the videotaped conversations. We slightly adapted the Rating Scales for the Assessment of Empathic Communication (REM, Nicolai et al., 2007), that is, we exchanged the term “doctor” from the original version with the term “interviewer.” The REM is a two-factorial instrument with six items measuring empathy and three items measuring confrontation (Nicolai et al., 2007). The confrontation scale measures the perception of the change recipient of being talked down or being admonished (e.g., “Did the interviewer admonish you?”; M = 1.49; SD = 0.66; α = 0.73). By contrast, the empathy scale measures the change recipients’ perception about the change agents’ empathic behavior (e.g., “the interviewer put himself/herself in my position”; M = 4.64; SD = 0.42; α = 0.79). Change recipients rated each of these items on a five-point Likert scale that was behaviorally anchored for each item (cf., Nicolai et al., 2007; e.g., 1 = not at all; 5 = a lot).

### Overview of Statistical Analysis

Change agents in both groups (MI and control) had a conversation about environmental behavior change with 1–3 recipients. This one-to-many design results in interdependence between change agents and change recipients, that is, one change agent is nested within several clients, which can result in biased statistical parameters (cf., Kenny et al., 2006). In order to allow an evaluation of unbiased statistical tests, we randomly selected a subsample of unique agent-recipient dyads. We report analyses for the interdependent dyads sample (*n* = 68) and the reduced sample of unique agent–recipient dyads (*n* = 26).

## Results

### Social and Cognitive Effects of MI

Our first hypothesis posited that MI in environmental feedback conversations reduces the amount of verbal threats by change agents. We used the intervention type (MI vs. control group) as independent variable and used *t*-tests for independent samples to compare the group means for MI non-adherent behavior of change agents. When considering the interdependent sample, change agents in the MI group showed significantly less observable MI non-adherent behaviors in comparison to change agents in the control group [*t*(17.05) = 3.96, *p* < 0.01, *d* = 0.83; see **Table 2**]. Whereas the rate of MI non-adherent behavior by change agents in the MI group was close to zero (M = 0.06), change agents in the control condition threatened change recipients’ about making changes at least two times within any 10-minute interval (M = 2.05). We replicated these findings in the unique dyads sample. This finding lends support to H1 by showing that MI indeed affects the social level of the interaction, in terms of the observable behavior of change agents.

**Table 2:** Comparisons of social and cognitive outcomes between the MI group and the control group.

	Interdependent dyads sample				Unique dyads sample			
	MI group	Control group			MI group	Control group		
Measure	M (SD)	M (SD)	*t*	Cohen’s *d*	M (SD)	M (SD)	*t*	Cohen’s *d*
Perceived confrontation (REM)	1.37 (0.54)	1.79 (0.85)	2.39*	0.65	1.24 (0.46)	1.85 (0.94)	2.18*	0.87
Perceived empathy (REM)	4.72 (0.33)	4.46 (0.57)	-1.98^†^	-0.52	4.71 (0.39)	4.36 (0.71)	-1.61	-0.64
Verbal threats (MI non-adherent)	0.06 (0.13)	2.05 (2.13)	3.96**	0.83	0.07 (0.13)	2.20 (2.62)	2.67*	1.06

MI, Motivational Interviewing; M, mean; SD, standard deviations are indicated in brackets; *p < 0.05; **p < 0.01; ^†^*p* < 0.05 (one-tailed).

Our second hypothesis stated that MI in environmental feedback affects change recipients’ cognitions about the interaction in terms of (a) increasing perceived empathy and (b) decreasing perceived confrontation. We calculated *t*-tests to compare group means between the MI and control group concerning perceived confrontation and perceived empathy. When considering the interdependent dyad sample, perceived confrontation and empathy were significantly different between the MI group and the control group (**Table 2**). Change recipients in the MI group perceived confrontation from change agents to a significantly lesser extent (M = 1.37) compared to change recipients in the control condition [M = 1.79; *t*(65) = 2.39, *p* < 0.05, *d* = 0.65]. Change recipients in the MI group also perceived higher empathy in comparison to change recipients in the control group [M = 4.72 vs. M = 4.46, *t*(22.95) = -1.98, *p* = 0.07, *d* = -0.52]. We replicated these results in the unique dyad sample, with the exception of perceived empathy which did not differ between groups.

Although there was no consistent difference on the perceived empathy, these findings mostly support H2 concerning positive effects of MI on change recipients’ social cognitions about the interaction.

### Interplay between Social Behavior and Cognitions

Our third hypothesis was that change agents’ social behavior and recipients’ perceptions of agents’ behavior are intertwined, such that the amount of verbal threats is positively related to perceived confrontation and negatively related to perceived empathy. To test this hypothesis, we calculated intercorrelations between perceived confrontation, perceived empathy, and MI non-adherent behavior by change agents. **Table 3** shows that the observed MI non-adherent behavior by change agents was strongly correlated with change recipients’ perceptions of being confronted (*r* = 0.54, *p* < 0.01). By contrast, there was a substantial negative correlation between MI non-adherent behavior and perceived empathy (*r* = -0.55, *p* < 0.01). In other words, an increase in MI non-adherent behavior was associated with an increased perception by change recipients that his/her change agent behaved in a confrontational manner. These results lend support to H3 concerning the social–cognitive interplay between the interaction behavior of change agents and the resulting cognitions in the respective change recipients. We also calculated these correlations separately for each group and found similar results. The rate of observed MI non-adherent behavior was positively correlated with perceived confrontation in MI dyads (*r* = 0.30, *p* < 0.05, *n* = 46) and control dyads (*r* = 0.76, *p* < 0.01, *n* = 18). Moreover, the rate of observed MI non-adherent behavior correlated negatively with perceived empathy both in MI dyads (*r* = -0.29, *p* < 0.05, *n* = 46) and control dyads (*r* = -0.66, *p* < 0.01, *n* = 18).

**Table 3:** Correlations between change agents’ verbal threats and change recipients’ cognitive variables.

	Interdependent dyads sample			Unique dyads sample		
	(1)	(2)	(3)	(1)	(2)	(3)
(1) Perceived confrontation (REM)	(0.73)^a^			(0.73)^a^		
(2) Perceived empathy (REM)	-0.65**	(0.79)^a^		-0.73**	(0.79)^a^	
(3) Verbal threats (MI non-adherent)	0.54**	-0.55**	(0.60)^b^	0.75**	-0.60**	(0.60)^b^

MI, Motivational Interviewing; REM, Rating scales for the assessment of empathic communication. **p < 0.01; ^a^Cronbach’s alpha; ^b^Intraclass correlation.

We replicated these findings in the unique dyad sub-sample, the direction and statistical significance of correlations were comparable (see **Table 2**). In the unique dyads sample, we also calculated correlations separately for each group (i.e., MI versus control group agents). Again, observed MI non-adherent behavior was positively correlated with perceived confrontation in MI dyads (*r* = 0.48, *p* = 0.068, *n* = 15) and control dyads (*r* = 0.78, *p* < 0.01, *n* = 11). Observed MI non-adherent behavior was negatively correlated with perceived empathy in MI dyads (*r* = -0.56, *p* < 0.05, *n* = 15) and control dyads (*r* = -0.63, *p* < 0.01, *n* = 11). These results support our third hypothesis that change agents’ social behavior and recipients’ perceptions of agents’ behavior are connected.

### Change Recipients’ Verbal Response to Threats in Social Interactions about Environmental Behavior Change

Our fourth and fifth hypotheses stated that verbal threats of change agents will be negatively related with change recipients’ expressed motivation (i.e., change talk; H4) and positively related with change recipients’ sustain talk (H5). To test these hypotheses, we calculated correlations between MI non-adherent behaviors by change agents and the respective verbal responses by change recipients (i.e., change talk or sustain talk). **Table 4** shows the results of these analyses separately for the interdependent and for the unique dyad sample. In both samples, MI non-adherent behaviors showed a substantial negative correlation with reasons to change (r = -0.40, *p* < 0.01). That is, the more change agents provided verbal threats to change environmental behavior, the less change recipients provided self-motivational statements such as reasoning why change might be beneficial for them. There were no significant associations between MI non-adherent behaviors and change recipients’ sustain talk measures. Moreover, although we did not hypothesize about linkages between perceived empathy and recipient behavior, we still explored linkages between these variables. It would be reasonable to expect that change agents’ empathy would address recipients’ need for relatedness. Hence, we conducted additional *post hoc* analyses using perceived empathy as an independent variable and recipients’ verbal responses (i.e., change and sustain talk) as dependent variables. **Table 4** shows that perceived agent empathy was positively related with reasons to change in both the unique dyad sample (*r* = 0.42, *p* < 0.01) and in the interdependent sample (*r* = 0.38, *p* = 0.053). In other words, the more change agents were perceived as empathic listeners that could relate to recipients’ problems, the more recipients showed intrinsically motivated behavior, in terms of stating reasons to change their environmental behavior.

**Table 4:** Intercorrelations between change agents’ verbal threats, empathy, and recipients’ verbal reactions.

		Interdependent dyads sample		Unique dyads sample	
	ICC	Verbal threats (MI non-adherent)	Perceived empathy (REM)	Verbal threats (MI non-adherent)	Perceived empathy (REM)
Change talk – Reasons	0.51*	-0.40**	0.42**	-0.42*	0.38^†^
Change talk – Activation	0.72**	-0.21	0.06	-0.18	-0.07
Change talk – Taking Steps	0.91**	0.12	0.14	0.02	0.02
Change talk – Commitment	0.85**	0.09	-0.15	0.07	-0.13
Sustain talk – Reasons	0.55**	-0.08	0.19	-0.14	0.21
Sustain talk – Activation	0.69**	-0.03	0.04	-0.12	0.21
Sustain talk – Taking Steps	0.36^†^	0.05	0.06	0.00	0.08
Sustain talk – Commitment	0.59*	-0.05	-0.18	-0.16	-0.17

Pearson’s correlations, two-tailed. MI, Motivational Interviewing; ICC, Intraclass correlation. **p < 0.01; *p < 0.05; ^†^*p* < 0.10.

### Effects of the MI Training Intervention on Environmental Attitudes and Intentions

We conducted separate two-factorial ANOVAs with the within-factor “time” (pre vs. post-conversation) and the between-factor “intervention type” (MI vs. control group) for the two dependent variables environmental attitudes and environmental intentions. Participants’ pro-environmental attitudes [*M*_0_ = 3.69; *M*_1_ = 3.80; *F*(1,66) = 5.16, *p* < 0.05, $\eta_{p}^{2}$ = 0.07] and pro-environmental intentions increased significantly over time [*M*_0_ = 3.43; *M*_1_ = 3.64; *F*(1,66) = 55.07, *p* < 0.01, $\eta_{p}^{2}$ = 0.46]. That is, conversations about pro-environmental behavior change positively affected environmental attitudes and intentions in both groups. We found no main effect for the factor “intervention type,” neither for the dependent variable environmental attitudes [*M*_MI_ = 3.75; *M*_Control_ = 3.74; *F*(1,66) = 0.01, *p* = 0.93, $\eta_{p}^{2}$ = 0.00] nor for the dependent variable environmental intentions [*M*_MI_ = 3.53; *M*_Control_ = 3.57; *F*(1,66) = 0.12, *p* = 0.73, $\eta_{p}^{2}$ = 0.00]. There was also no significant two-factorial interaction (time × intervention type), neither for environmental attitudes [*F*(1,66) = 0.84, *p* = 0.36, $\eta_{p}^{2}$ = 0.01] nor for environmental intentions [*F*(1,66) = 1.22, *p* = 0.27, $\eta_{p}^{2}$ = 0.02]. These findings from the interdependent dyad sample were replicated in the unique dyad sample.

Overall, these results indicate that the conversation about environmental behavior change positively affected participants’ environmental intentions and attitude. However, the improvement in environmental attitudes and intentions did not differ substantially between the MI conditions and the control group.

#### Environmental Action Plan

Finally, the number of planned environmental actions did not significantly differ between MI and the control group [*M*_MI_ = 3.51; *M*_Control_ = 4.16; *t*(66) = 1.2, *p* = 0.24, *d* = 0.32]. These findings were replicated in the unique dyad sample [*M*_MI_ = 3.67; *M*_Control_ = 4.00; *t*(24) = 0.52, *p* = 0.60, *d* = 0.21].

## Discussion

This study examined the cognitive and social reactions to potentially threatening conversations about pro-environmental behavior change. On the theoretical basis of SAT and SDT, we argued that individual feedback about environmental behavior (that is at odds with expected pro-environmental behavior) can pose a threat to individuals. Furthermore, social change agents can make these threats more salient by directly confronting or directing recipients toward a desired environmental behavior. According to SAT, participants use self-defense strategies to diminish such threats. Moreover, SDT suggests that threats harm the need of participants’ autonomy and may therefore diminish participants’ intrinsic motivation. In addition to examining the social and cognitive responses to potentially threatening feedback, we examined how feedback delivered using MI methods can help reduce perceived threats in social interactions.

Four main findings accrue from this study. First, MI affected the social level of interactions in terms of reducing change agents’ verbal behaviors that are potentially threatening to change recipients (i.e., confrontations, warnings, autonomy-restrictive behavior). Moreover, MI showed benefits for the cognitive level of interactions, in terms of increasing change recipients’ perceptions of a non-confrontational conversation.

Second, our findings illustrate the interplay between change agents’ verbal behaviors and recipients’ cognitions. Potentially threatening behaviors by change agents were negatively linked to recipients’ perceptions of empathy and positively linked to perceptions of being confronted.

Third, our results show how participants respond verbally to threats in social interactions. As hypothesized, verbal threats by change agents were negatively related to change recipients’ expressed motivation (i.e., verbal statements in favor of pro-environmental behavior change). Contrary to our expectations, however, verbal threats were not meaningfully related to change recipients’ sustain language (i.e., verbal self-defense strategies).

Fourth, the change recipients in our study experienced an improvement in pro-environmental attitudes and pro-environmental intentions as a result of the conversation, regardless of the condition (MI versus control group).

### Theoretical Implications

Our findings have several theoretical implications for research on the interplay of cognition and motivation in the face of threats during social interactions in general, and for research on environmental behavior change in particular. First, we argued that discussing discrepancies between a desired state (i.e., improving environmental behavior) and the current (often imperfect) state of environmental behavior can be potentially threatening (cf. Jonas et al., 2014). Drawing from SAT (Steele, 1988) and SDT (Deci and Ryan, 2000, 2002; Ryan and Deci, 2000, 2002), we argued how such threats within social interactions can negatively affect interactional dynamics. Our findings underscore theoretical linkages between SDT and MI as discussed in previous research (see e.g., Markland et al., 2005; Vansteenkiste and Sheldon, 2006; Leffingwell et al., 2007).

Second, our study offers empirical support for the beneficial cognitive effects of MI in dyadic interactions, as indicated by our finding that MI change agents were perceived as significantly less confrontational compared to change agents in the control group. This result is in line with the notion that “[o]ne ‘active ingredient’ in MI may simply be a decrease in unhelpful counselor responses” (Miller and Rollnick, 2013, p. 381). However, cognitions about being confronted with behavior change could make favorable behavior change on the part of the recipient less likely (e.g., Francis et al., 2005; Klonek et al., 2014). In order to account for these cognitions, we highlighted recipients’ perceptions of change agents’ verbal threats as a relevant aspect for understanding the influence of MI.

Third, our finding that verbal threats by change agents were meaningfully connected to recipients’ perceptions of behavioral threats aligns with previous findings from the medical field (Nicolai et al., 2007). However, our study extends this previous work in that we investigated the link between social behavior and cognitive appraisals of said behavior during environmental feedback conversations. Whereas medical feedback typically concerns problems of personal relevance, this does not necessarily apply to feedback regarding environmental behavior. Our findings thus contribute to the external validity of MI applications in the domain of environmental research and social interactions about environmental behavior. This is an important theoretical implication since MI is increasingly applied by environmental inspectors as a social change intervention (Forsberg et al., 2014). A review of the MI literature concluded that MI studies should also account for recipients’ perceptions of the interaction (Madson et al., 2009). In addressing this call, we integrated observer-based measures of behavioral threats and change recipient’s respective cognitive reactions.

Fourth, our findings shed light on the ways in which people respond to verbal threats in social interactions about environmental behavior change. That is, we compared both SAT and SDT in terms of their prediction for the underlying interactional mechanisms. SDT states that when individuals’ need for autonomy is threatened, they express less motivation for behavior change. Our results suggest that the core principle of SDT (Deci and Ryan, 2000, 2002; Ryan and Deci, 2000, 2002) finds practical implementation in the use of MI. The principle of autonomy is implemented by asking change agents to avoid confrontational behavior (e.g., Miller et al., 2004). The current study tentatively supports this SDT-based explanation of how MI might unfold its positive effect by showing that the amount of verbal confrontations was indeed negatively associated with change recipients’ expressed motivation, operationalized as verbally stated reasons to change. However, we found no negative relationships between verbal confrontations by change agents and other measures of change talk (i.e., activation, taking steps, commitment). Additional analyses revealed that empathy by change agents was positively related to change recipients’ motivational language (i.e., reasons to change). Although we abstained from formulating any hypotheses regarding this link, this result does align with the notion derived from SDT that change agents should address interactional partners’ need for relatedness in order to evoke intrinsic motivation. Moreover, because we calculated correlations, we can only conclude in terms of a general connection between the variables, instead of drawing directional conclusions.

Contrary to our expectations, our results do not align with the core tenet of SAT, which argues that threats to individuals’ self-integrity trigger defense responses such as using counter-arguments in order to restore their self-integrity. Our results showed no significant associations between change agents’ verbal threats and change recipients’ expressions of self-defense, operationalized as sustain talk. However, since this is the first study to investigate this relationship in the domain of environmental behavior, we would not necessarily discard the “threat results in defense” mechanism as posited in SAT. A meta-analysis of clinical studies in MI with over 1,000 participants reports a small (*r* = 0.07) but significant link between verbal threats (i.e., MI non-adherent behavior) and sustain talk (Magill et al., 2014). Our environmental behavior change intervention only supported the negative link between verbal threats and motivation to change (that was also reported by Magill et al., 2014). Our substantially smaller sample size and limited statistical power, in comparison to Magill et al. (2014), may have precluded us from establishing a significant relationship between threats and sustain talk. Moreover, verbal threats might affect recipients’ self-defense mechanisms in other ways, such as emotional and cognitive rather than verbal responses. This suggests a need for including measures of emotional and cognitive self-defense mechanisms in addition to observations of recipients’ verbal behavior in future research.

Finally, our findings indicate benefits for pro-environmental behavior not only in the MI condition, but also in the control group, suggesting an “any talk is good”-effect. However, our analyses also showed that the MI spirit of change agents in the control group was comparably high, presumably because control change agents also had a social science and psychology background. Miller and Rollnick (2013) argued that MI may benefit from a contrast effect, that is, MI will outperform social interaction based interventions that are mainly based on confronting and threatening change recipients to induce behavior change. Our control group condition may not have provided as extreme a contrast as previous studies comparing MI against confrontational therapeutic measures (e.g., in the treatment of alcohol addiction; see, e.g., Miller et al., 1993). Nevertheless, our findings lend themselves to implications for conversations about (environmental) behavior change in organizational practice.

### Practical Implications

Organizations are increasingly implementing social change agents in order to increase employees’ motivation for sustainable behavior (e.g., Kraft and Neubeck, 2004; Carrico and Riemer, 2011; Steiner et al., 2011; Kauran, 2013; Klonek and Kauffeld, 2015). For organizational practice, our findings imply that change agents aiming to improve environmental conservation behaviors need to show interaction behavior that adheres to MI principles and aligns with the core tenets of SDT (i.e., change agents should respect recipients’ need for autonomy and facilitate self-determined decisions). For example, an environmental intervention study by Werner et al. (2012, p. 419) used social change agents to promote energy-saving behavior and reported that agents “did not explicitly ask students to commit to turning off lights, but did ask if students were “on board” and willing to help [.] (...) [W]e did not want to use a stronger request that might feel coercive to students.”

The authors reported that the conversational intervention outperformed a condition in which a sign (i.e., sticker) reminded change recipients to conserve energy. Our study adds implications for communicative interventions (or socio-interaction based interventions) by highlighting the need for change agents to respect recipients’ need for autonomy, in line with SDT.

To assess whether this need is being met in change agent/recipient interactions, organizations could implement quality assurance measures (e.g., the MI spirit rating scale from the MITI). Finally, in order to reduce perceived confrontation during such interactions, organizations should consider training change agents in MI (see also Klonek and Kauffeld, 2015). Discussing discrepancies between current energy use and organizational benchmarks to save a certain amount of energy would likely not only be perceived by employees as less threatening but could also yield the intended behavior change as a result.

### Limitations and Future Directions

We acknowledge several limitations of this study. First, we gathered a student sample rather than studying environmental change agents and recipients in the field. However, it could certainly be argued that graduate students should show conscious environmental behavioral conduct similar to employees of organizations. Future research should aim to replicate our results in a field setting, although this may posit several challenges. Participants are often reluctant to allow video recordings in the field (Spee and Jarzabkowski, 2011; Meinecke and Lehmann-Willenbrock, 2015) and change agents do not always succeed in recording their conversations (Forsberg et al., 2014). Nevertheless, future studies should investigate the association of verbal threats and recipients’ response in field settings and with larger sample sizes in order to substantiate our results.

Second, our measure of recipients’ motivation to change showed somewhat limited reliability, such that the results for this measure should be interpreted cautiously. Previous research on observed change and sustain talk in clinical settings has reported similarly low ICCs for recipients’ verbal codes (e.g., Baer et al., 2008; Gaume et al., 2008; Magill et al., 2010). Future research should strive to improve the reliability of observing recipients’ motivation for change and further refine the coding instrument, perhaps by adapting it to certain features of the specific interaction context (in our case, pro-environmental conversations).

Third, whereas we obtained both social and cognitive measures, we did not include self-reports to measure recipients’ motivation. Our decision to focus on observations of their verbal behavior was guided by prior research in MI. Following this approach allowed us to draw comparisons between our findings and previous research on MI in clinical settings (for an overview, see Magill et al., 2014). Moreover, previous research from the clinical field indicates that observational measures of motivation for or against change (e.g., observed resistance) have greater predictive value for behavior change compared to self-reported motivation measures, which is likely due to the elimination of self-representation bias when using observational measures (Westra, 2011). Future research should investigate whether this applies to the area of environmental change intervention as well. Moreover, based on our study, we cannot draw conclusions about actual behavior change. However, it would be interesting to assess how the use of MI in conversations about environmental behavior change might influence recipients’ actual environmental behavior. Future research should strive to include follow-up measures that allow conclusions about actual change in behavior and lifestyle resulting from conversations where MI is applied.

Fourth, this study has shed light on the psychosocial and cognitive effects of MI in dyadic conversations about environmental behavior change. However, we did not consider the neural mechanisms that may be associated with threats and change language during social interactions. The analysis of a neuronal mechanism via the use of functional magnetic resonance or electroencephalography during face-to-face interactions is methodologically still very challenging. Nonetheless, there is preliminary evidence that change and sustain talk of change recipients have a neuronal correlate (Feldstein Ewing et al., 2011, 2014; Houck et al., 2013). Moreover, a recent neurocognitive study by Feldstein Ewing et al. (2014) emphasized that self-motivating change talk has to be generated from the change recipient rather than simply by repeating change-related statements. This underscores the core idea behind MI that motivation to change is created via the social-interactive component of behavioral change interventions (Feldstein Ewing et al., 2014).

Fifth, the sample size of participants in the MI group was twice as large as participants in the control group. This implies larger SE in the control group and reduced power to detect significant differences between the two groups. Moreover, because change agents talked on average with three clients, interdependence between dyads should be considered. However, given our small sample of change agents, the analysis of multi-level models would result in biased parameter estimates (Maas and Hox, 2005). Therefore, we reran all analyses using a unique dyad sample. Future studies should incorporate larger change agent samples in order account for statistical interdependence by testing for actor effects, partner effects, and relationship effects.

Finally, whereas our study examined MI skills in the context of dyadic interactions about environmental behavior, future research should also investigate how MI skills can be implemented in change interventions aimed at the group or community level. For instance, one study has successfully applied MI for changing water disinfection practices within communities in Zambia (Thevos et al., 2000). Future research should examine how MI can facilitate environmental education that targets a broader audience, for example for increasing environmental engagement within an entire municipality.

In sum, this paper offers the following contributions: First, we integrated theoretical perspectives of SAT and SDT in order to investigate how participants respond to threats in social interactions. Next, we identified MI as a fruitful communication approach to reduce threats in social interactions and to promoting more favorable social and cognitive reactions in conversations about environmental behavior change. We tested this approach in a field experiment and tested how actual verbal threats and the perception of those threats differed in dyads in which an MI approach is used, in contrast to a control group. Our findings showcase the influence of interactional dynamics on both social and cognitive responses to threats during conversations about environmental behavior. We show how behavioral observations can provide a non-obtrusive measurement of motivation during conversations about behavior change. No study to date has assessed change recipients’ perceptions and observational language measures during a conversation about environmental behavior change with change agents who use MI. We addressed this research gap while also adding to the few studies that have already investigated the use of MI in the context of environmental behavior (Tribble, 2008; Forsberg et al., 2014). We discussed theoretical implications of our findings in the context of SAT and SDT and for organizational practice, particularly concerning environmental behavior change initiatives. Moreover, we demonstrated the value of a multi-method approach that integrates subjective perceptions and observational measures for gaining insights into the conversational dynamics surrounding perceived threats in social interactions and for untangling the interplay between interactional partners’ behaviors and cognitions.

## Conclusion

This study provides support for the benefits of MI in terms of reducing actual verbal expressions of threats as well as alleviating negative perceptions of threats during social interactions. By connecting both the social and the cognitive level of conversational partners, our study contributes to a deeper understanding of people’s responses to threats in social interactions.

## Conflict of Interest Statement

The authors declare that the research was conducted in the absence of any commercial or financial relationships that could be construed as a potential conflict of interest.

## Footnotes

1 We will use the more generic terms ‘change agent’ throughout this paper for individuals that promote behavior change (e.g., therapists, physicians, advisors, environmental inspectors, block leaders) and ‘change recipients’ for individuals that carry out behavior change (e.g., clients, patients, conversational partners, environmental inspectors, employees).
