# Supplementary material for: Using Motivational Interviewing to reduce threats in conversations about environmental behavior
Source: Front Psychol. 2015 Jul 21;6:1015. doi: 10.3389/fpsyg.2015.01015 (PMC4508486; doi:10.3389/fpsyg.2015.01015)
Supplement: Supplementary file 1 [file Table_1.DOCX]

**Appendix A**

Interview guide used for interviewers in the MI group during the interview.

| Interview guide  **Introduction**   1. **Step: Saying thank you.**   - Showing appreciation the conversational partner’s for participation.   1. **Step: Meeting the conversational partner where he/she is.**   e.g., „What brings you in today?”  - Conversational partner gets the chance to explain „his“/“her“ perspective  - As change agent: use reflective listening!   1. **Step: What is the perspective of the conversational partner with respect to his/her environmental behavior**   e.g., „How is your typical energy consumption distributed over the week?” or “Which environmental resources do you use?”  - As change agent: use reflective listening!  **FEEDBACK**   1. **Step: Personalized feedback about environmental behavior (ecological footprint)**   -Obtain the conversational partner’s perspective: e.g., „It says here that you waste a lot of water. What do you think about that?“  -As change agent: use reflective listening!  **EVOCATION**   1. **Step: Pros and Cons (Decisional balance)**   -Provide decisional balance for environmental behavior (this can refer to a single field, e.g. saving energy or less driving etc.)  *Pros of changing*   - - - „Which pros does ... (e.g. saving energy …) have for you?“ or „What do you like about the idea of living environmentally friendly?”     - Summarizing pros.   *Cons of changing*   - - - „Which cons does ... (e.g. saving energy …) have for you?“ or „What would   you add?“   - - - Summarizing cons.  1. **Step: Querying extremes**  - e.g. „What are the worst things that might happen if you don’t make this change?“ or „What is the best thing that might happen if you do make this change?“   - - *Exploring goals and values in life* (do environmental values, goals regarding our society, or responsibility towards nature and animals come up arise?)     - *What is important to for you in life?*     - *What kind of person would you like to be?*     - *Importance- or Confidence-Ruler*   „On a scale where 0 is not important at all and 10 is extremely important, how important is it to you to change _____?”  “What needs to happen that you move from ___ to a ____ [higher number]? “  “On a scale where 0 is not confident at all and 10 is extremely confident, how confident are you that you could ____? Explain why you rated yourself at a___ and not at a 0?”  **Commitment Plan / Action Plan**   1. **Step: Summarize + NEXT Steps**   *Action plan*  „So, what do you think you will be doing next?”  “In which way would you like to change your behavior?”  “What is the next step?“  „What are you going to do in the next 2 days?”  “Have you ever taken one of these steps before to realize this goal?”   1. **If there is no decision yet:**  - Investigate if your conversational partner is stuck in his ambivalence (empathic perspective taking). What might help them to make a decision? |
| --- |


**Appendix B**

Instructions used for interviewers in the control group as preparation before the interview.

| Task | Convince your conversational partner to show more pro-environmental behavior. |
| --- | --- |
| Steps | **Before the interview:**   1. Review the questionnaire of your conversational partner and mark for yourself in which environmental areas your conversational partner shows little pro-environmental behavior. 2. Please note that all the answers that your conversational partner has checked and which contain an "i" in the code must first be reversed. I.e., the reply is in the opposite direction of the statement.   **During the interview:**   1. Give feedback to your conversational partner concerning the areas in which he/she is not environmentally conscious. 2. Convince your conversational partner to improve his/her environmental awareness. You can use the “Interview guide” for help. |
| Time | 1. Reviewing your conversational partner’s questionnaire (preparation): 10 minutes 2. Interview with conversational partner: 30-45 minutes |
| Resources | - Questionnaire of conversational partner’s environmental behavior. - Interview Guide - Tips and advice on how to improve environmental behavior |

**Appendix C**

Interview guide used for interviewers in the control group during the interview.

| **Interview Guide**  **1) Setting the agenda**  *“In this interview, we want to speak about your environmental behavior. This means what you do or can do to treat our planet in an ecologically sustainable way. Therefore, you have filled out a questionnaire that has given us a good impression and conveys what you should do differently…”*  **2) Asking about current environmental behavior**  *“What is your typical energy consumption spread across the week? What environmental resources do you use?”*  **3) Giving feedback over energy consumption / ecological footprint**  *The general rules of the questionnaire:*  *When a question was checked off at the lowest level, there was really nothing to optimize. But when questions were checked off at a higher level... here, the client should plan specific measures to change something.*  *Questions that are marked with „i“ must be reversed. That is, if marked at the lowest level, participants can change something about their environmental behavior.*  *You can use the sheet “Tips for improving environmental behavior” that lists how your conversational partner can help the environment.*  **4) Asking for measures to increasing pro-environmental behavior**  **5) Setting up an intervention plan / Give tips and advice on how to improve environmental behavior.** |
| --- |

**Appendix D**

Sheet with tips and advice used by interviewers in both groups

| **Tips and advice on how to improve environmental behavior**   1. Reduce the consumption of meat. 2. Switch to green energy supplier. 3. Use energy-saving light bulbs instead of conventional ones. 4. At the end of the work day, turn off all devices by switching off the power strip from the network. 5. Only heat when it is absolutely necessary. 6. Ventilate only for short times (i.e., allow fresh air to circulate through the room). 7. If possible, only use a Notebook for work. 8. Be sure to switch the lights off every time you leave the office. 9. When you plan to be away, shut down the computer and turn off the power switch. 10. Keep coffee you drink warm in a thermos. 11. Climbing stairs keeps you fit and saves power. 12. Reduce traveling by plane. 13. Eat locally produced food. 14. Purchase second-hand products. |
| --- |
